# Effectiveness of Fewer Falls, an online group-based self-management fall prevention programme for people with multiple sclerosis: protocol of a randomised controlled trial

**DOI:** 10.1136/bmjopen-2024-089217

**Published:** 2025-01-06

**Authors:** Susanna Tuvemo Johnson, Maria Flink, Kristina Gottberg, Elizabeth Walker Peterson, Ulrika Meijer, Johanna Bylinder, Marie Kierkegaard, Charlotte Ytterberg

**Affiliations:** 1 Department of Neurobiology, Care Science and Society, Karolinska Institutet, Stockholm, Sweden; 2 Department of Women's and Childrens Health, Uppsala University, Uppsala, Sweden; 3 Women's Health and Allied Health Professionals Theme, Karolinska Institutet, Stockholm, Sweden; 4 Department of Occupational Therapy, University of Illinois Chicago, Chicago, Illinois, USA; 5 Academic Specialist Center, Stockholm, Region Stockholm, Sweden

**Keywords:** Self-Management, Behavior, eHealth, Multiple sclerosis, Patient Participation

## Abstract

**Introduction:**

*Fewer Falls* is a manualised self-management fall prevention programme co-developed for and with ambulatory and non-ambulatory people with multiple sclerosis (PwMS). Findings from a feasibility study indicate the necessity of a full-scale randomised controlled trial (RCT).

**Methods and analysis:**

A parallel-group RCT with a mixed methods process evaluation as well as a cost-effectiveness evaluation will be conducted. We aim to recruit 240 PwMS, who will be stratified by ambulation level and randomised 1:1 in blocks of eight to intervention or control. The group-based self-management fall prevention intervention involves eight 2-hour online synchronous sessions (approximately eight participants/group) facilitated by a licensed healthcare professional and home assignments to be completed by participants between sessions. The setting is online, and participants can be located anywhere in Sweden. The control and intervention groups will also receive a brochure on fall risk factors and fall prevention in addition to their standard MS care and rehabilitation. Data collection will be performed at baseline and 3, 6 and 12 months after the start of the intervention. Falls will be monitored via a short message service every week during 1 year from the start of the intervention. The primary outcome is fall frequency (falls/person/year). Secondary outcomes include injurious falls, falls control, fear of falling, falls self-efficacy, activity curtailment, perceived effect of MS, self-rated health and cost-effectiveness.

**Ethics and dissemination:**

Ethical approval has been obtained from the Swedish Ethical Review Authority (registration numbers 2022-06667-01 and 2023-07723-02). The RCT will adhere to the Declaration of Helsinki. Written consent to participate will be obtained from all participants. Study-related information about participants will be stored securely at Karolinska Institutet. The results will be presented in peer-reviewed journals, through the patient organisation Neuro Sweden, at conferences, and in social media.

**Trial registration number:**

ClinicalTrials.gov, NCT05789225.

**STRENGTHS AND LIMITATIONS OF THIS STUDY**

- This study protocol describes an online group-based, self-management fall prevention intervention featuring group dialogue and individual action plans for both ambulatory and non-ambulatory people with multiple sclerosis (PwMS).
- The comprehensive process evaluation will yield understanding of implementation and the mechanisms of impact for the intervention.
- The health economic evaluation will provide unique knowledge as no study has evaluated the cost-effectiveness of fall prevention programmes in PwMS.
- The online delivery of the intervention may exclude PwMS with low computer/technical skills and low socioeconomic status.

## Introduction

 Multiple sclerosis (MS) is a chronic inflammatory, demyelinating and neurodegenerative disease that typically is progressive in nature.^1^ Several symptoms of MS are known fall risk factors, including impaired balance, gait and cognition.^2 3^ In people with MS (PwMS), approximately 56% experience a fall within 3 months.^4^ Those PwMS who have experienced a fall in the past year have an 82% probability of falling again within 6 months and a 56% probability of an injurious fall.^5^ Falls among PwMS are associated with fear of falling, low health-related quality of life and injuries.^3 6^ Because PwMS frequently experience injurious falls,^7^ the socioeconomic costs for both PwMS and society are potentially high. Surprisingly, there are no studies on costs related to falls in PwMS. However, existing research describing the cost of falls for other patient populations with neurological problems ^8 9^ have suggested the high healthcare costs associated with falls among PwMS.

Despite the recognition that several MS symptoms influences fall risk,^2 3^ most fall prevention interventions for PwMS only address physical impairments. There is a major gap in existing evidence regarding behavioral and environmental influences on fall risk in PwMS. The importance of comprehensive approaches to fall prevention targeting modifiable risk factors through fall prevention programmes for PwMS has been highlighted.^10^ Another major gap in MS falls research is the scarcity of studies that focus on falls among the 25% of the MS population who can walk only a few steps or not at all, ie, are non-ambulatory.^6^ Fall prevention interventions for non-ambulatory PwMS show promising results but are rare.^11 12^ Although ambulatory and non-ambulatory PwMS share the same fall risk factors,^2 13^ new evidence suggests that some fall risk factors are unique to non-ambulatory PwMS.^13^ Thus, fall prevention programmes for PwMS must target the unique needs and multifactorial fall risks of PwMS, with consideration that members of the target audience may be ambulatory or non-ambulatory and typically will experience fluctuations in functional status and disease over time.

To address the wide variety of fall risk factors for diverse patient populations, research has highlighted the benefits of self-management for PwMS.^10^ Self-management has been defined as ‘the individual’s ability to manage the symptoms, treatment, physical and psychosocial consequences, and lifestyle changes intrinsic in living with chronic conditions’.^14^ Self-management is especially important for PwMS due to the daily fluctuations in functioning and the unpredictable nature of the disease. PwMS have identified support for self-management as one of their top 10 research priorities.^15^ Despite the importance of self-management, few randomised controlled trials (RCTs) have evaluated self-management fall prevention programmes for PwMS,^16^,^18^ and none of those trials included definitions of self-management. A scoping review^19^ identified that few RCTs did not favour either intervention or control groups, which displays the importance of further research.

To address the diverse influences of fall risk factors for both ambulatory and non-ambulatory PwMS, RCTs that evaluate the effectiveness of well-described self-management fall prevention interventions are needed. We have therefore used a co-design methodology and developed the online group-based fall prevention intervention *Fewer Falls* together with PwMS and healthcare professionals^20 21^ following the Medical Research Council recommendations for complex interventions.^22^ A feasibility study has been performed,^23^ indicating feasible results (in manuscript). The online synchronous format was decided based on the suggestion of PwMS that online meetings would make participation in the programme possible even when energy levels are low.^21^ Online programmes and applications are becoming more common,^24 25^ as they can involve geographically spread participants at lower costs.^26^ A thorough description of the *Fewer Falls* programme development process has been published.^21^

### Aim and research questions

This study aims to evaluate the effectiveness of the self-management fall prevention programme *Fewer Falls* on falls in PwMS. The hypothesis is that the fall frequency in the *Fewer Falls* intervention group will be lower than that in the control group.

Research questions (RQs):

RQ 1a: Is there a difference in fall frequency between the intervention group and the control group 6 months (primary endpoint) after the start of the intervention?

RQ 1b: Is there a difference in the fall frequency between the intervention group and the control group 12 months (sustainability) after the start of the intervention?

RQ 2: Are there differences in secondary outcomes between the intervention group and the control group?

RQ 3: How do process evaluation components (context, implementation and mechanisms of impact) inform the interpretation of outcomes?

RQ 4: What is the cost-effectiveness of *Fewer Falls* 12 months after the start of the intervention?

## Methods and analysis

### Design

The study will use a parallel-group RCT design with a mixed methods process evaluation as well as a health economic evaluation. The trial is registered at ClinicalTrials.gov (NCT05789225), and any updates to the protocol will be registered.

This study protocol adheres to the Standard Protocol Items: Recommendations for Interventional Trials (SPIRIT) guidelines for reporting intervention trials.^27^

**Figure 1:**
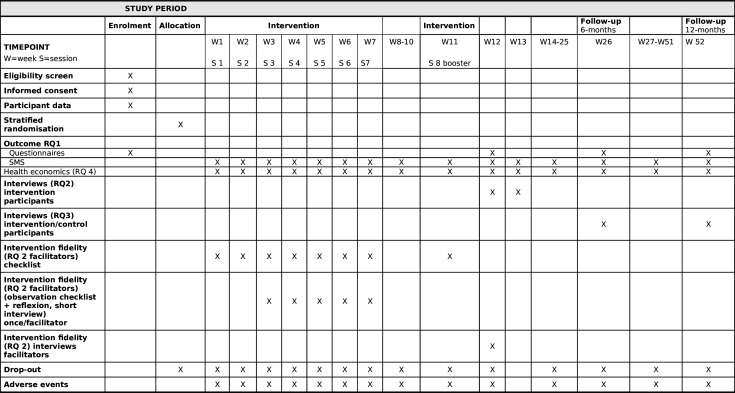
Overview of the study. Schedule of enrolment, allocation, intervention and assessments. RQ = research question

**Figure 2:**
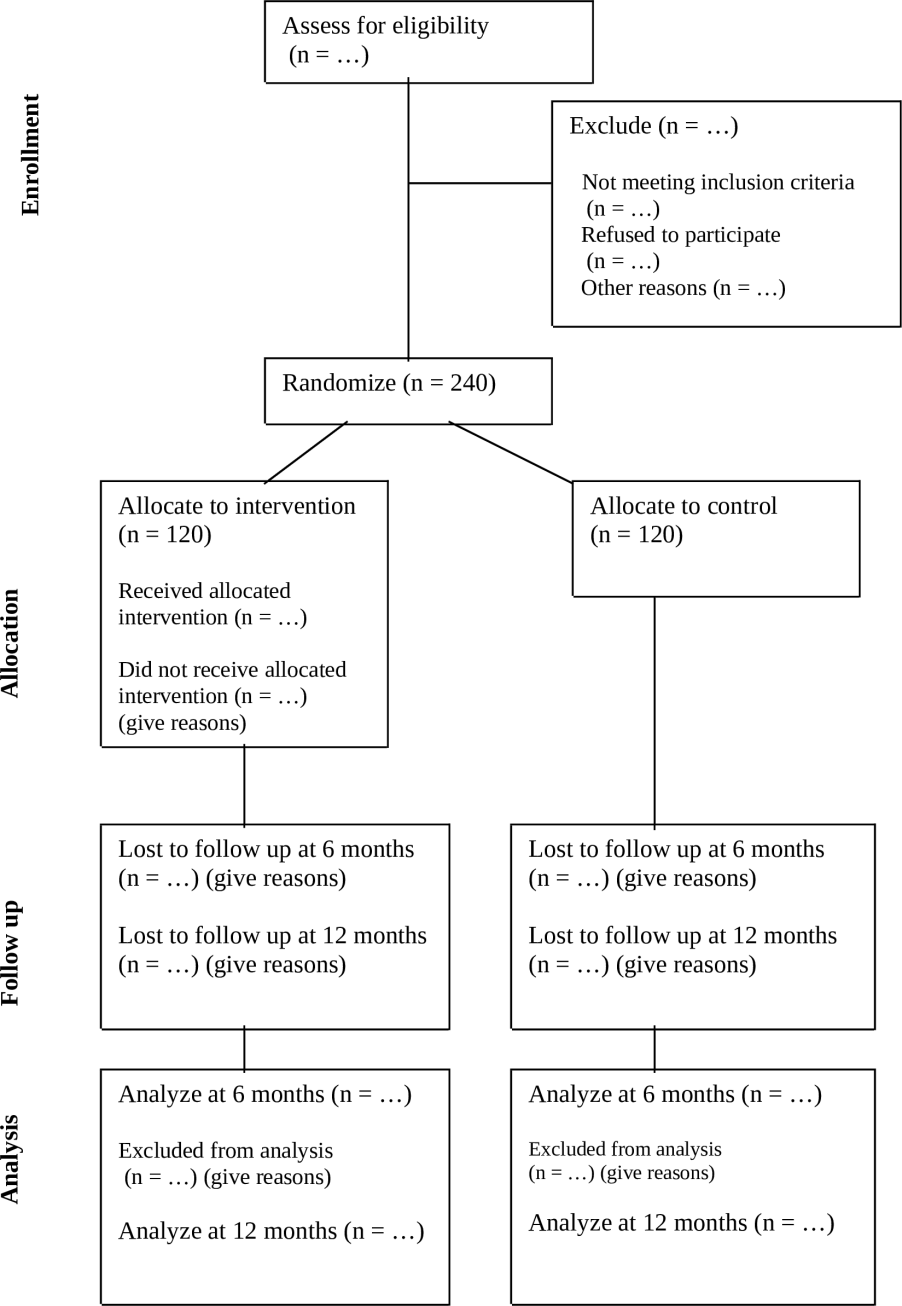
CONSORT diagram showing the flow of participants through each stage of the trial.

Figure 1 displays the study enrolment, intervention and assessments. The study flow chart is presented in figure 2.

### Patient and public involvement

The intervention was co-developed in close collaboration with PwMS and healthcare professionals through an iterative process. The intervention development process has been previously described,^21^ PwMS representatives from the patient organisation Neuro Sweden have collaborated as project partners in all stages of the study.

### Participants

The inclusion criteria for participation are self-reported diagnosis of MS; age ≥18 years; ability to walk or independently transfer from bed to chair/wheelchair with or without aids, but without assistance of another person (corresponding to ˂7.5 on the Expanded Disability Status Scale); experienced one or more falls during the last year; ability to understand and communicate in Swedish; and access to and self-rated ability to use technical devices for online meetings and falls data submission, that is, computers or tablets with internet access and mobile phones to receive and send short message service (SMS).

The statistical power calculation for the primary outcome, fall frequency, is based on data from our feasibility pilot RCT study (in manuscript). The pilot study included 45 participants, randomised into an intervention or control group. Falls were monitored weekly via SMS for 18 weeks from the start of the intervention. Data from 27 participants who at baseline reported a fall within the previous 3 months were used. Based on the findings of 2.45 falls for the intervention group (n=11) versus 3.69 in the control group (n=16) over a period of 18 weeks, we determined that a sample size of 84 participants per group is necessary to achieve adequate statistical power (0.8, alpha=0.05).^28^

Because an online publication on interventions for people with chronic conditions reported a high attrition and drop-out rate,^29^ we aim to recruit up to 240 participants, allowing for up to a 35% drop-out rate. The sample size is based on a Poisson distribution, which aligns with the nature of our primary outcome falls/person/year.

### Study setting

Programme content is delivered by the intervention facilitator, that is, group leader via synchronous online sessions. Between sessions, participants will be asked to complete assignments in their home environment. The assignments are designed to encourage participants to practise skills learnt during the intervention, that is, to carry out their individual action plans. The participants and intervention facilitators may be located anywhere in Sweden.

### Recruitment and procedures

Participants will be recruited via flyers placed in inpatient and outpatient MS and rehabilitation clinics, from social media advertisements published by Karolinska Institutet, and by the patient organisation Neuro Sweden.

The notices will include a link to a webpage with study information and an online template where interested individuals can fill in their contact information. A research assistant with training in Good Clinical Practice^30^ will screen the completed templates regularly, contact potential participants by phone or e-mail (depending on PwMS’ choice of contact), and provide potential participants with information about the study. Individuals expressing interest in the study will be screened for eligibility. The informed consent process will be initiated with PwMS who meet the eligibility criteria. Specifically, eligible PwMS will be sent written information. Informed consent (online supplemental material) is obtained through BASS, an integrated online treatment and assessment platform. The platform is provided by the eHealth Core Facility at Karolinska Institutet, Stockholm, Sweden. Individuals providing informed consent will be included.

Baseline assessment will be supported by the use of the BASS database. The PwMS will subsequently be randomised to the intervention group or the control group using a data-generated system (Sealed Envelope Ltd. 2021). Participants will be stratified as non-ambulatory (able to walk a maximum of 5 m with assistance or not able at all) or ambulatory in a 1:1 allocation ratio of blocks of eight. Participants will be contacted by phone to inform them about group allocation.

Recruitment began in December 2023, and the expected completion of the study is January 2027. An overview of the study schedule of enrolment, allocation, intervention and assessments are presented in figure 1.

### Control

Participants allocated to the control group will receive a brochure about fall risk factors and fall prevention for PwMS in addition to their standard MS care and rehabilitation.

### Intervention

The intervention group participants will receive (a) the same brochure about fall risk factors and fall prevention strategies for PwMS provided to the control group participants, (b) the opportunity to participate in the *Fewer Falls* programme and (c) their standard MS care and rehabilitation. The manualised *Fewer Falls* programme features self-management content created to enhance the participant’s ability to manage fall risks in everyday life. The intervention is considered a complex intervention^22^ as it includes multiple components, behaviours, expertise and skills both by PwMS and group leaders and permits flexibility in its components. Through *Fewer Falls*, participants learn to (a) identify their fall risk, (b) make own action plans and follow out the planned activities, (c) be aware of their capacity in relation to expectations and demands in daily life, (d) to follow-up on the action plan and (e) know about behavioural change and how to sustain their motivation to prevent falls.

The manualised intervention consists of eight online synchronous (Zoom Video Communications) sessions: seven 2-hour sessions conducted weekly, and one 2-hour booster session conducted 4 weeks after the seventh session. The participants will receive home assignments to perform between the sessions. The assignments are designed to encourage participants to practice skills learnt during the intervention, ie, to carry out their individual action plans. Each session has the following structure: check-in, follow-up on home assignments, interactive presentation with discussions in smaller groups and/or the whole group, introduction to home assignment and check-out (table 1).

**Table 1:** Main topic and activities of *Fewer Falls*

Session*/Home assignment#	Main topics and activities
Session 1: Acquaintance with group, programme and technique.	Check-in.Presentation: Programme overview.Presentation/Discussion: How to use the online platform, get to know each other and build trust.Check-out.
Home assignment 1.	Participants post a short introduction on the online platform (BASS).
Session 2: Falls in MS.	Check-in.Discussion: Group consensus on group interaction.Interactive presentation: Why do people with MS fall?Presentation: Introduction to the action plan to prevent falls.Interactive presentation: New behavioursCheck-out.
Home assignment 2.	Participants take a photo of a fall hazard they want to address and describe how the image in the photo presents a fall risk. Start individual action plan no. 1.
Session 3: My fall risks.	Check-in.Follow-up: Discussion on each other’s fall risks.Interactive presentation: Environmental fall risks. Working with action plan no. 1.Check-out.
Home assignment 3.	Participants carry out activities planned in individual action plan no. 1.
Session 4: Behavioural change.	Check-in.Follow-up: Evaluation of action plan no. 1.Interactive presentation: Behavioural change to prevent falls including supportive and hindering factors related to falls prevention behaviour change.Start of writing action plan no. 2.Check-out.
Home assignment 4.	Participants evaluate action plan no. 1 and complete the writing of action plan no. 2 and start the planned activities.
Session 5: Thoughts and emotions and fall risk.	Check-in.Follow-up: Presentation of and reflection on action plan no. 2.Interactive presentation: How can thoughts and emotions affect the risk of falling?Check-out.
Home assignment 5.	Participants carry out activities planned and evaluate action plan no. 2.
Session 6: Physical symptoms and fall risk.	Check-in.Follow-up: Evaluation of action plan no. 2.Interactive presentation: Physical factors related to fall risks and how these risks can be reduced.Start of writing action plan no. 3.Check-out.
Home assignment 6.	Participants complete the writing of action plan no. 3 and start the planned activities.
Session 7: Aids to reduce fall risk.	Check-in.Follow-up: Evaluation of action plan no. 3.Interactive presentation: How can aids reduce fall risk?Interactive presentation: Behaviour changes and maintenance.Check-out.
Home assignment 7.	Participants carry out and evaluate action plan no. 3. Write, carry out and evaluate action plan no. 4. Declaration of interest to stay in touch.
Session 8: Booster session.	Check-in.Follow-up: Evaluation of action plan nos. 3 and 4.Programme evaluation.Check-out.

* Group leader led synchronous online group- sessions, #individual home- assignments between sessions.

The intervention is delivered to groups of approximately eight participants. A health professional (ie, a physiotherapist, occupational therapist or social worker) recruited via the researchers’ network facilitates the online synchronous group sessions. Seven group leaders will be recruited to lead one or two cycles (ie, seven sessions followed by one booster session) of the programme in concurrently ran groups. The research team recognise the importance of involving several group leaders to allow for effective assessment of programme fidelity and reduce the possibility that successful programme outcomes would be attributed to the skill set of one or two group leaders. Approximately 15 cycles of the *Fewer Falls* programme will be run. The group leaders will not be involved in the usual care of the participants.

The group leaders will complete an extensive online training programme before delivering *Fewer Falls*. This training programme will consist of asynchronous online modules. Collectively, these modules (a) describe the aim, structure, content and theoretical foundation of *Fewer Falls* as well as the role of the group leaders; (b) provide an overview of MS, falls and fall risk factors for PwMS; (c) orient group leaders to the *Fewer Falls* group leader manual; and (d) provide detailed descriptions of content and process of *each programme* session. Each module of the training programme ends with a quiz or a reflective question. The final module of the training programme consists of a synchronous online role-play session. In addition, the group leaders will receive instructions on how to use the digital tools used during the *Fewer Falls* programme.

To ensure participant adherence to the programme, the group leaders will contact participants who missed one session. If participants miss two sessions in a row, the research assistant will contact the participants.

### Theoretical basis, self-management approach and pedagogical model

The leading theory informing the *Fewer Falls* programme content and process is the Social Cognitive Theory (SCT).^31 32^ Reciprocal determinism, the central concept of SCT, refers to the dynamic and reciprocal interaction of person (with learnt experiences), environment (external social context) and behaviour (responses to stimuli to achieve goals).^32^ Reciprocal determinism is applied in *Fewer Falls* because participants, with their respective experiences, work together to learn about fall risks and management strategies and change their behaviours to reduce their risk of falling. The SCT components of observational learning and reinforcements align well with the programme’s focus on group format; participants are learning and receiving feedback from peers. According to SCT, self-regulation refers to the ability to understand and manage own behaviours.^32^ In *Fewer Falls*, self-regulation supporting the reduction of fall risk is fostered through using two to four action plans. Through each action plan, participants identify and explain a specific fall risk, a strategy to address that fall risk, their level of confidence in implementing that strategy, the deadline for completion and resources to support their success in implementing the strategy. Each action plan includes a section for participants to evaluate the extent to which the planned strategy was implemented. Participants are given the opportunity to report on the status of their action plans and work together to refine action plans as needed. Self-efficacy, a central component in SCT, is described as a possible mechanism by which self-management is achieved.^33^ An important goal of *Fewer Falls* is to build participants’ falls self-efficacy.

Lorig and Holman’s operationalisation of self-management^33^ was carefully applied to develop *Fewer Falls*. Specifically, the programme includes discussions about self-management tasks: medical management of MS symptoms that can reduce fall risk; role management, for example, PwMS’ role as partner, parent and/or employee; and emotional management with emphasis on thoughts and emotions in relation to falls. The depth and focus of the discussions are dependent on participants’ interests/priorities (eg, identified fall risks) and experiences. Lorig and Holman described six self-management skills, all of which are included in *Fewer Falls* to foster participants’ ability to manage fall risks in daily life: problem solving, decision making, resource utilisation, patient-provider partnership, self-tailoring and the aforementioned action planning.

SCT is complemented by the Transtheoretical Model (TTM) for behaviour change^34^ as a theoretical basis for *Fewer Falls*. TTM is primarily applied through an interactive discussion led by the group leaders on the six phases of health behaviour change (precontemplation, contemplation, preparation, action, maintenance of behaviour and relapse) that participants may experience as they learn to use action plans to prevent falls.

**Figure 3:**
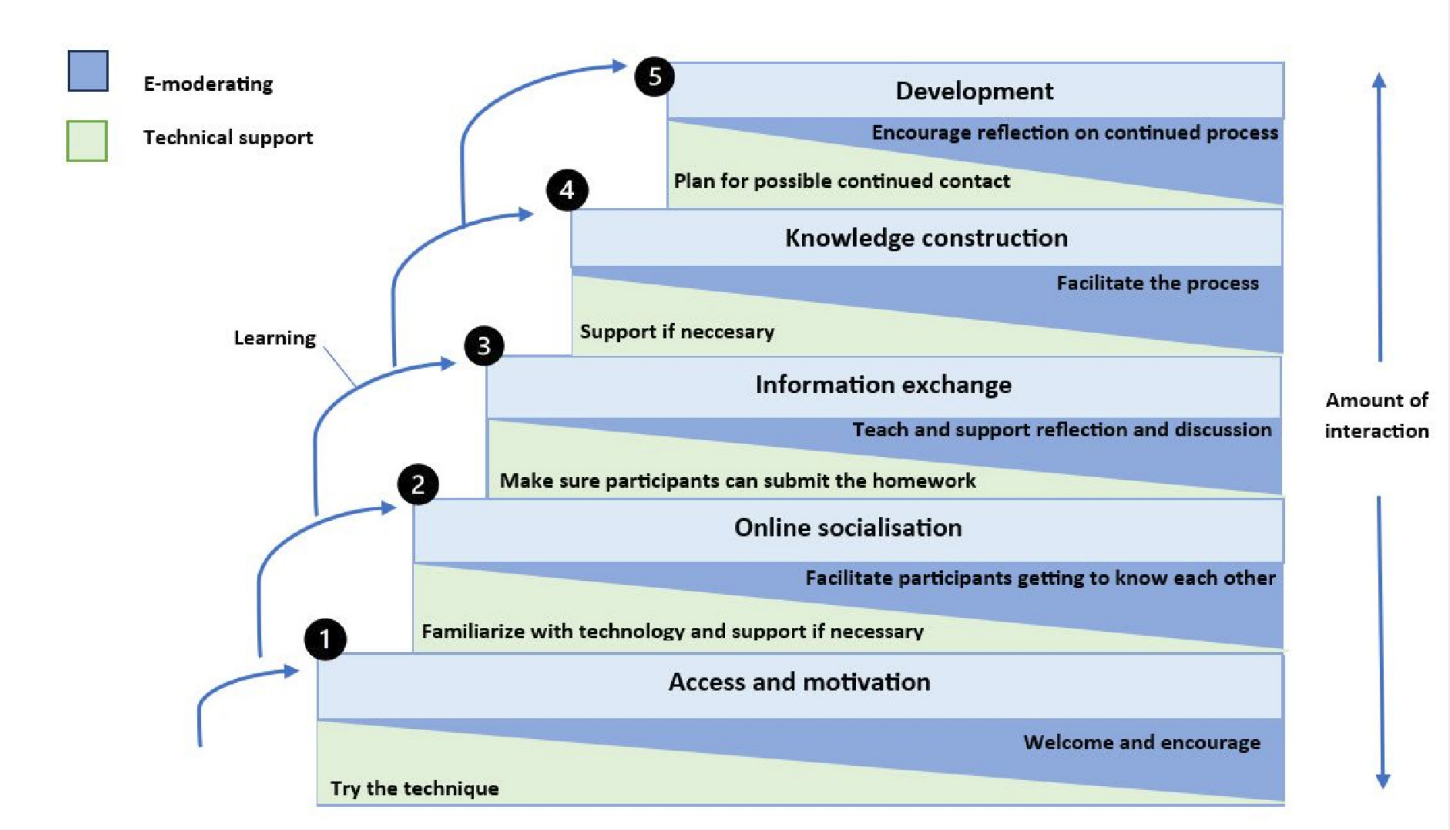
The five-stage model as applied in *Fewer Falls*

To guide the online learning component of *Fewer Falls,* the Salmon 5 Stage Model was utilised because it was specifically developed to support e-learning in groups.^35^ The model consists of five stages: (1) access and motivation, (2) online socialisation (team building), (3) information exchange, (4) knowledge construction (critical thinking) and (5) development (review and responsibility for own learning). The amount of interactivity is increased in each stage of the model. The model stresses the importance of group leaders acting as moderators/facilitators of online discussions. In the early stages of the programme, group leaders support participants’ motivation and engagement with the rest of the group. The model also recommends that facilitators taper off their leadership as the course progresses, allowing space for greater peer support and peer learning.^35^ The application of the model as used in *Fewer Falls* is presented in figure 3.

### Data collection and outcome measures

#### RQ 1a, RQ 1b and RQ 2: effectiveness study

Data will be collected using the digital platform BASS database. The data collection will be blinded as the participants receive a link to the BASS database, which provides them with access to the questionnaires that are completed online. Baseline data includes the sociodemographic information (ie, age, sex, civil status, work status and educational level); disease-related characteristics (ie, MS severity, time since diagnosis, symptoms, medications and other treatments, other diagnosis and aids); formal/informal care; cognitive function (collected through the short version of the Montreal Cognitive Assessment Scale^36^); anxiety and depression (collected through the Hospital Anxiety and Depression scale^37^); fatigue (assessed by the Fatigue Severity Scale^38^); health literacy (assessed via the Swedish version of the Health Literacy Survey European Questionnaire/16 item version^39^); self-reported falls and fall related injuries in the previous 6 months; and expectations on the programme.

The primary outcome of *Fewer Falls* is fall frequency.^40^ The primary endpoint is 6 months after the start of the intervention. However, falls will be monitored in both intervention and control groups from the start of the *Fewer Falls* programme until 12 months after the start of intervention, as recommended in fall prevention literature.^41^ An online SMS will be sent to the intervention and control participants once a week asking, ‘Have you fallen within the last week?’ The total number of falls, number of fallers and injurious fall incidence^40^ will be recorded. Participants answering ‘yes’ to the weekly SMS will receive a questionnaire through BASS that includes a series of questions about fall-related injuries. A fall is defined as ‘an unexpected event in which the participants come to rest on the ground, floor or lower level’.^41^ Secondary outcomes, collected at baseline and 3, 6 and 12 months after the start of intervention, are sense of control of falls prevention assessed by the Falls Control Scale^42^ (administered to all participants), a direct measure of fear of falling^43^ and falls self-efficacy assessed by the Short Falls Efficacy Scale-International^44^ (administered to ambulatory participants), the Spinal Cord Injury Falls Concern Scale^45^ (administered to non-ambulatory participants) and a revised version of the Short Falls Efficacy Scale-International^46^ (administered to all participants). The following are also administered to all participants to collect data: activity curtailment assessed by a single question,^43^ perceived impact of MS assessed by the Multiple Sclerosis Impact Scale^47^ and self-rated health assessed by the EuroQol-5D-5L.^48^

Participants who discontinue the intervention will be contacted and asked if they are willing to continue responding to the SMS and questionnaires.

#### RQ 3: process evaluation

Consistent with the Medical Research Council guidelines,^22 49^ the process evaluation will focus on contextual factors (eg, online delivery of the programme), implementation (the implementation process, fidelity, dose, adaptations and reach) and mechanisms of impact (participant responses to and interactions with the intervention).

Data will be collected from all group leaders before and after the training programme, after each session and after the end of the *Fewer Falls* programme. Data collection will include structured questions on group leader background (eg, sex, age and profession), perceived preparedness in facilitating the group and fidelity to programme manual, and semi-structured interviews. The semi-structured interviews conducted after the training programme and after the last session of the intervention will cover group leaders’ perspective on experiences of participation, contextual factors, implementation and mechanisms of impact. The interviews will be conducted face-to-face or through Zoom.

Process evaluation data will also be yielded from intervention group participants. Specifically, data will be collected from a purposively selected sample of intervention group participants (n=20–30) to represent variation in, for example, age, sex and ambulation level. Semi-structured individual interviews will be conducted by a research team member via telephone or Zoom within approximately 3, 6 and 12 months after the start of the intervention. The interviews will explore the experiences of the study participants, contextual factors and mechanisms of impact. Data from the programme platform will be collected on intervention group participants’ online activities, for example, the action plans. Participants who discontinue the intervention will be contacted and asked if they are willing to participate in a short interview to understand their reasons for not completing the programme.

#### RQ 4: health economic evaluation

Participants answering ‘yes’ to the weekly SMS inquiring about falls experienced in the last week will receive a questionnaire through the BASS database with questions on fall-related injuries, that is, healthcare utilisation, municipal care utilisation, sickness absence, medications, support from significant others and need for technical aids, including walking aids.

Estimations of resources needed to deliver the *Fewer Falls* programme will be made. Group leaders’ involvement, which includes participating in the group leader training and subsequently preparing for and delivering the *Fewer Falls* programme, will be the main programme expense. To estimate the monetary value of resource use, both for participants in the RCT and for resources needed to conduct the intervention, cost data will be retrieved from the cost per patient database held at the Swedish Association of local authorities and regions, regional price lists, as well as from Statistics Sweden. Data on quality-adjusted life years (QALYs) will be measured using the EQ-5D-5L at all timepoints.

#### Adverse events

All adverse events associated with the trial will be discussed and recorded.

### Analysis

#### Data management and statistical methods

##### RQ 1a, RQ 1b and RQ 2: effectiveness study

Intention-to-treat and per-protocol analyses will be performed. Per-protocol analyses will include participants who fulfil content adherence, defined as completion of at least two action plans.

Descriptive statistics will be used to present data, for example, means, medians, frequencies, SD, quartiles and counts. Drop-out analyses of baseline characteristics will be explored with parametric and non-parametric analyses depending on the data level.

The primary analysis for falls (falls/person/year) will be conducted using generalised linear mixed models (GLMM) with a Poisson link function to account for count data distribution and potential overdispersion. The model will include fixed effects for the intervention group and random effects for participant variations over time. Interactions between variables will be included if deemed necessary. Linear mixed model analyses will be used for normally distributed secondary outcomes. For categorical outcomes, GLMM with logistic or ordinal regression will be used, depending on the nature of the outcome (binary or ordinal). The primary endpoint is 6 months after the start of the intervention. In addition, analyses of sustainability of potential effects will be performed after 12 months. Any missing data will be handled using appropriate methods, such as multiple imputation, to ensure robustness in analyses. Analyses will be performed and reported in accordance with the Consolidated Standards of Reporting Trials (CONSORT) guidelines for RCT.^50^ A data management plan is established and stored at the Research Data Office at Karolinska Institutet.

##### RQ 3: process evaluation

RQ 3: Qualitative content analysis will be used for interview data, and descriptive statistics will be used for quantitative data. Data from the group leaders and participants will be analysed separately.

##### RQ 4: health economic evaluation

The PwMS participating in the *Fewer Falls* programme will be compared with the control group using intention-to-treat analysis. Appropriate statistical models will be used to estimate changes in resource use over time, considering the distribution of the data collected. Incremental costs will be estimated and compared between the intervention group and the control group. QALYs will be used as the primary health outcome in the economic evaluation, estimated from EQ-5D-5L data. Analyses will be performed and reported in accordance with the Consolidated Health Economic Evaluation Reporting Standards (CHEERS) guidelines for health economic evaluations.^51^

## Ethics and dissemination

This study has ethical permission from the Swedish Ethical Review Authority (registration no 2022-06667-01 and 2023-07723-02), and the procedures will be performed in accordance with the Declaration of Helsinki. Information will be given orally and in writing, and all participants will give their signed informed consent before the study will start. The results will be published in peer-reviewed journals, at research conferences and meetings, through the patient organisation Neuro Sweden and in social media. Deidentified data will be available on reasonable request after publication of the results.

## Discussion

The evaluation of the *Fewer Falls* programme is an important advancement in fall prevention research because findings will yield insights regarding the effectiveness of an intervention designed to address the specific needs of a unique patient population (ie, ambulatory and non-ambulatory PwMS). This protocol directly addresses the imperative stated by Tuvemo Johnson *et al*^19^ by defining and operationalising ‘self-management’ to support efforts to explain how the intervention activities relate to established frameworks of self-management and mechanisms of impact. *Fewer Falls* is based on theories and incorporates a self-management approach, which will help PwMS to identify and manage their individual multifactorial fall risks.

Previous RCTs of self-management fall prevention programmes^16^,^18^ designed for PwMS have not shown intervention superiority compared with control. However, none of these were developed using a co-design process with PwMS and/or healthcare professionals. Thereto, none of the programmes were grounded in self-management theories regarding programme activities. *Fewer Falls* is based on, and all components of the programme are linked to, theories of self-management. This will potentially support PwMS to identify, understand and self-manage their multifactorial fall risks, for example, by using their individual action plans.

The fact that the intervention is delivered online adds to the uniqueness of the programme. To our knowledge, *Fewer Falls* is the only fall prevention intervention in Sweden that uses an online platform to deliver synchronous and asynchronous programme content. Available evidence suggests that online delivery will support recruitment and programme implementation, including reach, by reducing barriers to programme access.^52^ Importantly, online delivery eliminates the need to travel to the site where the programme is being delivered, thus increasing programme availability across Sweden. Access to specialised MS care and rehabilitation in Sweden is largely available only in larger cities. Thus, online delivery has great potential to contribute to more equal access to fall prevention programmes and, thus, support healthcare equality in our country. In addition to eliminating travel-related expenses and logistics, the online intervention will require less time for/effort associated with travelling and therefore may reduce fatigue associated with programme participation.

However, the study has some limitations. The programme is directed to PwMS in Sweden with technical and cognitive ability to participate in a complex intervention. The online delivery will exclude PwMS without technical devices for online meetings and those without technical and cognitive ability to use such devices. Further, unstable network connectivity for PwMS and group leaders may cause short or long outages, which can affect the programme delivery and outcome. In addition, PwMS with impaired communication abilities and skills might not benefit from group discussions. Finally, data including outcomes are mainly self-reported, which should be kept in mind when interpreting the forthcoming results.

In line with the Medical Research Council recommendation for the development and evaluation of complex interventions,^22^ throughout the project, we have collaborated with PwMS and healthcare professionals in the development of the intervention.^20 21^ The described project involves outcome and process evaluations, as well as a health economic evaluation. Programme effectiveness will be evaluated using a full-scale powered RCT, which is unique to self-management fall prevention research involving both ambulatory and non-ambulatory PwMS. In addition, falls will be monitored up to 12 months, as recommended.^41^

The comprehensive process evaluation will enhance understanding of implementation strengths and challenges and the mechanisms of impact for the intervention. The latter is limited in fall prevention research and an important contribution to self-management fall prevention research. As no study has evaluated the cost-effectiveness of fall prevention programmes in PwMS, the health economic evaluation will contribute with unique insights. Our extensive level of collaboration with stakeholders throughout previous and present phases of the project is original and highly salient to the ultimate benefit for end users.

## supplementary material

10.1136/bmjopen-2024-089217 online supplemental file 1

## References

[1] Walton C, King R, Rechtman L (2020). Rising prevalence of multiple sclerosis worldwide: Insights from the Atlas of MS, third edition. Mult Scler.

[2] Gunn HJ, Newell P, Haas B (2013). Identification of risk factors for falls in multiple sclerosis: a systematic review and meta-analysis. Phys Ther.

[3] Coote S, Comber L, Quinn G (2020). Falls in People with Multiple Sclerosis: Risk Identification, Intervention, and Future Directions. Int J MS Care.

[4] Nilsagård Y, Gunn H, Freeman J (2015). Falls in people with MS--an individual data meta-analysis from studies from Australia, Sweden, United Kingdom and the United States. Mult Scler.

[5] Cameron MH, Thielman E, Mazumder R (2013). Predicting falls in people with multiple sclerosis: fall history is as accurate as more complex measures. Mult Scler Int.

[6] Rice L, Kalron A, Berkowitz SH (2017). Fall prevalence in people with multiple sclerosis who use wheelchairs and scooters. Medicine (Baltimore).

[7] Cameron MH, Asano M, Bourdette D (2013). People With Multiple Sclerosis Use Many Fall Prevention Strategies but Still Fall Frequently. Arch Phys Med Rehabil.

[8] Minet LR, Peterson E, von Koch L (2020). Healthcare Utilization After Stroke: A 1-Year Prospective Study. J Am Med Dir Assoc.

[9] Weir S, Samnaliev M, Kuo T-C (2018). Short- and long-term cost and utilization of health care resources in Parkinson’s disease in the UK. *Mov Disord*.

[10] Gunn H, Endacott R, Haas B (2018). Development of a balance, safe mobility and falls management programme for people with multiple sclerosis. Disabil Rehabil.

[11] McArthur AR, Peterson EW, Sosnoff J (2023). Online Delivery of the Individualized Reduction of Falls Intervention for Persons With Multiple Sclerosis Who Use a Wheelchair or Scooter Full-time: A Pilot Study. Int J MS Care.

[12] Rice LA, Yarnot R, Sung J (2022). Pilot Study of a Fall Prevention and Management Intervention Program for People With Multiple Sclerosis Who Use a Wheelchair or Scooter Full-Time. *Arch Rehabil Res Clin Transl*.

[13] Rice LA, Sung J, Peters J (2018). Perceptions of fall circumstances, injuries and recovery techniques among power wheelchair users: a qualitative study. Clin Rehabil.

[14] Barlow J, Wright C, Sheasby J (2002). Self-management approaches for people with chronic conditions: a review. Patient Educ Couns.

[15] National Institute for Health and Care Research (2023). James lind alliance top 10s priorities for research. https://www.jla.nihr.ac.uk/top-10-priorities.

[16] Chanes DC, Piza FM de T, San Martin G (2021). Fall prevention education for people with multiple sclerosis: a randomized clinical trial. Int J Qual Health Care.

[17] Cameron MH, Hildebrand A, Hugos CL (2022). Free From Falls education and exercise program for reducing falls in people with multiple sclerosis: A randomized controlled trial. Mult Scler.

[18] Cattaneo D, Gervasoni E, Pupillo E Educational and Exercise Intervention to Prevent Falls and Improve Participation in Subjects With Neurological Conditions: The NEUROFALL Randomized Controlled Trial. Front Neurol.

[19] Tuvemo Johnson S, Flink M, Peterson E (2023). Self-management of falls in people with multiple sclerosis: A scoping review. Clin Rehabil.

[20] Meijer U, Flink M, Tuvemo Johnson S (2024). Preventing falls in multiple sclerosis: a qualitative study on user requirements for a self-management programme. *Disabil Rehabil*.

[21] Tuvemo Johnson S, Ytterberg C, Peterson E (2024). Development of Fewer Falls in MS-An Online, Theory-Based, Fall Prevention Self-Management Programme for People With Multiple Sclerosis. Health Expect.

[22] Skivington K, Matthews L, Simpson SA (2021). A new framework for developing and evaluating complex interventions: update of Medical Research Council guidance. BMJ.

[23] Kierkegaard M, Peterson E, Tuvemo Johnson S (2022). Online self-management fall prevention intervention for people with multiple sclerosis: a feasibility study protocol of a parallel group randomised trial. BMJ Open.

[24] Kannan M, Hildebrand A, Hugos CL (2019). Evaluation of a web-based fall prevention program among people with multiple sclerosis. Mult Scler Relat Disord.

[25] Mazuz K, Biswas S, Lindner U (2020). Developing Self-Management Application of Fall Prevention Among Older Adults: A Content and Usability Evaluation. *Front Digit Health*.

[26] Murgado-Armenteros EM, Torres-Ruiz FJ, Vega-Zamora M (2012). Differences between Online and Face to Face Focus Groups, Viewed through Two Approaches. J theor appl electron commer res.

[27] Butcher NJ, Monsour A, Mew EJ (2022). Guidelines for Reporting Outcomes in Trial Protocols: The SPIRIT-Outcomes 2022 Extension. JAMA.

[28] Gu K, Ng HKT, Tang ML (2008). Testing the ratio of two poisson rates. *Biom J*.

[29] Meyerowitz-Katz G, Ravi S, Arnolda L (2020). Rates of Attrition and Dropout in App-Based Interventions for Chronic Disease: Systematic Review and Meta-Analysis. J Med Internet Res.

[30] (2024). Good clinical practice. https://kliniskastudier.se/english/training/good-clinical-practice-gcp.

[31] Bandura A (2004). Health Promotion by Social Cognitive Means. *Health Educ Behav*.

[32] Bandura A (1986). Social foundations of thought and action: a social cognitive theory.

[33] Lorig KR, Holman H (2003). Self-management education: history, definition, outcomes, and mechanisms. Ann Behav Med.

[34] Prochaska JO, Velicer WF (1997). The transtheoretical model of health behavior change. *Am J Health Promot*.

[35] E-Moderating SG (2011). The key to online teaching and learning.

[36] Nasreddine ZS, Phillips NA, Bédirian V (2005). The Montreal Cognitive Assessment, MoCA: a brief screening tool for mild cognitive impairment. J Am Geriatr Soc.

[37] Zigmond AS, Snaith RP (1983). The hospital anxiety and depression scale. Acta Psychiatr Scand.

[38] Krupp LB, LaRocca NG, Muir-Nash J (1989). The Fatigue Severity Scale: Application to Patients With Multiple Sclerosis and Systemic Lupus Erythematosus. Arch Neurol (Chicago).

[39] Bergman L, Nilsson U, Dahlberg K (2023). Validity and reliability of the swedish versions of the HLS-EU-Q16 and HLS-EU-Q6 questionnaires. BMC Public Health.

[40] O’Malley N, Coote S, Staunton FM (2023). A core outcome set for evaluating the effectiveness of mixed-diagnosis falls prevention interventions for people with Multiple Sclerosis, Parkinson’s Disease and stroke. PLoS One.

[41] Lamb SE, Jørstad‐Stein EC, Hauer K (2005). Development of a Common Outcome Data Set for Fall Injury Prevention Trials: The Prevention of Falls Network Europe Consensus. *J American Geriatrics Society*.

[42] Tennstedt S, Howland J, Lachman M (1998). A Randomized, Controlled Trial of a Group Intervention to Reduce Fear of Falling and Associated Activity Restriction in Older Adults. J Gerontol Ser B: Psychol Sci Soc Sci.

[43] Clemson L, Kendig H, Mackenzie L (2015). Predictors of injurious falls and fear of falling differ: an 11-year longitudinal study of incident events in older people. J Aging Health.

[44] Kempen GIJM, Yardley L, van Haastregt JCM (2008). The Short FES-I: a shortened version of the falls efficacy scale-international to assess fear of falling. Age Ageing.

[45] Butler Forslund E, Roaldsen KS, Hultling C (2016). Concerns about falling in wheelchair users with spinal cord injury--validation of the Swedish version of the spinal cord injury falls concern scale. Spinal Cord.

[46] Yardley L, Beyer N, Hauer K (2005). Development and initial validation of the Falls Efficacy Scale-International (FES-I). Age Ageing.

[47] Hobart J, Lamping D, Fitzpatrick R (2001). The Multiple Sclerosis Impact Scale (MSIS-29): A new patient-based outcome measure. Brain (Bacau).

[48] Herdman M, Gudex C, Lloyd A (2011). Development and preliminary testing of the new five-level version of EQ-5D (EQ-5D-5L). Qual Life Res.

[49] Moore GF, Audrey S, Barker M (2015). Process evaluation of complex interventions: Medical Research Council guidance. BMJ.

[50] Schulz KF, Altman DG, Moher D (2010). CONSORT 2010 statement: updated guidelines for reporting parallel group randomized trials. Obstet Gynecol.

[51] Husereau D, Drummond M, Augustovski F (2022). Consolidated Health Economic Evaluation Reporting Standards 2022 (CHEERS 2022) statement: updated reporting guidance for health economic evaluations. BMJ.

[52] Van Denend T, Peterson EW, McArthur AR (2022). A process evaluation of an on-line fall prevention and management program for individuals who use wheelchairs or scooters living with multiple sclerosis. Front Public Health.

