# Supplementary material for: Effectiveness of Fewer Falls, an online group-based self-management fall prevention programme for people with multiple sclerosis: protocol of a randomised controlled trial
Source: BMJ Open. 2025 Jan 6;15(1):e089217. doi: 10.1136/bmjopen-2024-089217 (PMC11749722; doi:10.1136/bmjopen-2024-089217)
Supplement: online supplemental file 1 [file bmjopen-15-1-s001.docx]

# Information for research participants

## We invite you to participate in a research project. This document provides details about the project and what participation entails.

**What is the project and why do we want you to participate?**

Falls and fear of falling are common among people with multiple sclerosis (MS). In collaboration with persons with MS and healthcare professionals, we have developed a self-management program to prevent falls. We now aim to evaluate the program’s effectiveness. Since you have shown interest in the study, we are inviting you to participate.

The principal researcher for this project is Karolinska Institutet. Research principal means the organization responsible for the project. The application has been approved by the Ethics Review Authority, with diary numbers 2022-06667-01 and 2023-07723-02.

## How is the project conducted?

As a participant in the project, you will be asked to answer questions about your health and life situation. You will then be randomly assigned to either a control group or a treatment group. Participants in the control group will receive a leaflet with information about falls and fall risk in MS. Participants in the treatment group will participate in the self-management program, which includes eight group online meetings. Each group will consist of approximately eight people with MS and will be led by a group leader. The first seven meetings will occur weekly, and the final meeting takes place four weeks later. Each session will last about two hours. Between meetings, you will have homework, estimated to take up to two hours per week. The program focuses on learning strategies to reduce the risk of falls in everyday life. You will share experiences and create action plans with other participants and the group leader.

Participants from the control group and treatment group will complete questionnaires on four occasions: before group assignment, immediately after, and at 6 and 12 months after the start of the control or treatment period. You may also be asked to participate in one or two individual interviews. Completing the questionnaires is estimated to take up to one hour, and each interview about 45 minutes. During the 12-month study period, you will receive a weekly SMS asking if you have experienced a fall. If you have, you will be asked to provide details about the circumstances. All questionnaires will be conducted online and the interviews will be conducted online or via telephone.

## Possible consequences and risks of participating in the project

Participation in the study does not involve any medical risks or discomfort. However, participants in the treatment group may experience fatigue due to the intensity of the program. Sharing your experiences of living with MS and falls may cause emotional discomfort. Individual conversations are available during and between group meetings.

You can withdraw from the study at any time.

## What happens to my data?

## The project will collect and register information from you through questionnaires about your life situation (e.g., age, education level, work situation, and need for aids at home) and your health. The health questionnaire includes questions about your MS, daily activities, and how you manage fall risk in your everyday life. You will also receive a weekly SMS asking, “Have you fallen?” If you answer yes, you will be asked additional questions about the fall.

## All data will be coded, meaning your name or social security number will not appear on any documents when reporting the study results. All documents will be kept as journal records in locked areas, protected by confidentiality regulations according to the Publicity and Confidentiality Act, ensuring no unauthorized person can access the information.

## Karolinska Institutet, 171 77 Stockholm, +46 8 524 80 000, is responsible for your personal data. According to the EU’s data protection regulation, you have the right to access the information about you handled in the project free of charge and to have any errors corrected if necessary. You can also request that your information be deleted and that the processing of your personal data be restricted. However, the right to erasure and to limit the processing of personal data does not apply when the data is necessary for the current research.

## If you have any questions, please contact the principal researcher, Charlotte Ytterberg, Karolinska Institutet, NVS, Department of Physiotherapy, Alfred Nobels allé 23, 141 83 Stockholm, +46 8 524 888 82. The Data Protection Officer can be reached at dataskyddsombud@ki.se, +46 8 524 864 73. If you are dissatisfied with how your personal data is processed, you have the right to lodge a complaint with the Swedish Data Protection Authority, the supervisory authority.

## How do I receive information about the results of the project?

If you are interested in accessing your collected data or the overall results of the study, please feel free to contact the project manager. There is no obligation to review the study results.

## Insurance and compensation

If you choose to participate in the study, you will be covered by Karolinska Institutet's group insurance with Kammarkollegiet: Special personal injury insurance (SPS) 2016.

No compensation is paid for participation in the study.

## Participation is voluntary

Your participation is voluntary, and you can withdraw at any time. If you choose not to participate or decide to discontinue, you do not need to provide a reason, and it will not affect your future care or treatment.

If you wish to withdraw, please contact the project manager (see below).

## Principal researcher

Charlotte Ytterberg, docent

charlotte.ytterberg@ki.se

+468-524 888 82

Karolinska Institutet

Department of Neurobiology, Care Sciences and Society

Division of Physiotherapy

Alfred Nobels Allé 23

141 83 Huddinge, Sweden

# Consent to participate in the project

I have received oral and/or written information about the study and have had the opportunity to ask questions. I get to keep the written information.

- I agree to participate in the project Evaluation of a self-management program to prevent falls in people with multiple sclerosis.

| Place and date | Signature |
| --- | --- |
|  |  |
|  | Name clarification |
|  |  |
