# Supplementary material for: Effectiveness of Fewer Falls, an online group-based self-management fall prevention programme for people with multiple sclerosis: protocol of a randomised controlled trial
Source: BMJ Open. 2025 Jan 6;15(1):e089217. doi: 10.1136/bmjopen-2024-089217 (PMC11749722; doi:10.1136/bmjopen-2024-089217)
Supplement: Supplementary file 2 [file bmjopen-2024-089217.reviewer_comments.pdf]

PEER REVIEW HISTORY

BMJ Open publishes all reviews undertaken for accepted manuscripts. Reviewers are asked to complete a checklist review form and are provided with free text boxes to elaborate on their assessment. These free text comments are reproduced below.

ARTICLE DETAILS

Title (Provisional)

Effectiveness of Fewer Falls, an online group-based self-management fall prevention program for people with multiple sclerosis: protocol of a randomized controlled trial

Authors

Tuvemo Johnson, Susanna; Flink, Maria; Gottberg, Kristina; Walker Peterson, Elizabeth; Meijer, Ulrika; Bylinder, Johanna; Kierkegaard, Marie; Ytterberg, Charlotte

VERSION 1 - REVIEW

**Reviewer**	**1**
**Name**	**Mazuz, Keren**
**Affiliation**	**HAC**
**Date**	**07-Sep-2024**
**COI**	**no interests**

Thank you for the opportunity to review the manuscript. The topic is timely and important, but it requires major revisions, especially since there is no dedicated results section, and the discussion needs substantial improvement. Below are my detailed suggestions:

1. Clearly distinguish between patient experience and medical objectives, including how effectiveness and cost-effectiveness are determined.

2. Add a hypothesis to the manuscript.

3. Clarify the participant group—those who have experienced one or more falls in the past year. How does this group differ from those who have never fallen?

4. Clarify whether age is treated as an independent variable.

5. Include more literature about online programs, not just regarding falls and the illness condition. For example, refer to studies like https://pubmed.ncbi.nlm.nih.gov/34713024/.

6. The role of the leader who facilitates and motivates participants should be evaluated in real-time. Is this feasible? Additionally, evaluate the impact of a leader on the effectiveness of online or self-management programs.

7. Provide more details on the socio-demographic characteristics of the participants.

8. Update Table 1 to include information on synchronous versus asynchronous meetings, group versus individual settings, and whether a leader is involved.

9. Clarify the use of models for program evaluation or for analyzing data and outcomes.

10. How often are participants interviewed during the 8-week period?

11. Discuss whether the cost of the program is covered by health insurance.

12. Expand the discussion section—it currently reads like a summary and is only a page and a half long.

13. The Results section is missing; what were the key findings?

14. Clarify the meaning of "ambulatory" and "non-ambulatory" in the context of the ability to walk.

15. There is no feasibility survey of online evaluations. Consider including one.

**Reviewer**	**2**
**Name**	**Cameron, Michelle**
**Affiliation**	**Oregon Health & Science University**
**Date**	**11-Sep-2024**
**COI**	**I have carried out, and received funding for, studies of fall-prevention programs for people with multiple sclerosis.**

Thank you for this submission. I am excited to see ongoing efforts to develop and evaluate fall prevention interventions for PwMS. However, this manuscript describing your proposed protocol would be made stronger with some changes.

Please clarify the aims of this study. Are you studying the effectiveness of the intervention and/or its cost-effectiveness? In some places the cost-effectiveness is emphasized (e.g. cost is mentioned in the very first paragraph of the introduction and in the first sentence of the aim and research questions) but in other places cost is not mentioned (e.g. the abstract, the overall hypothesis).

I find the hypothesis as stated, "that after participating in Fewer Falls, PwMS will identify and self-manage risk factors for falls in their everyday life, and thereby reduce the number of falls." somewhat unclear and difficult to test. This hypothesis would require four things to be true: 1) PwMS identify their fall risk factors in everyday life, 2) self-manage fall risk factors in everyday life, 3) reduce their number of falls, 4) that 1 and 2 are causative of 3. Furthermore, the research questions (RQ) do not directly address these issues. RQ1 addresses the number of falls, i.e. #3. 1, 2 and 4 are not addressed by the RQs, and then the RQs address

completely different things i.e. secondary outcomes (RQ2), process evaluation components (RQ3), and cost effectiveness (RQ4).

It is not clear to me if this study is ongoing or planned at this time. Either way, I think one could align the aims, hypothesis(es), and research questions with each other in this manuscript. If the study is planned it could probably be improved. If it is ongoing one can still improve this description.

In the methods please add some specific details regarding patient and public involvement. You state that "the intervention has been developed in close collaboration with PwMS and healthcare professionals." Please be specific e.g. the intervention was developed initially by the investigators based on their background knowledge and current literature. An initial version of the intervention was presented to a focus group of 5 PwMS (some demographics about them) and 3 healthcare professionals (a nurse, a PT and an OT). Based on this input the intervention was modified ... The readership needs this level of detail to understand the degree of influence of various parties.

Regarding the participants, how did you/will you verify they have MS? or that they have had one or more falls in the past year?

Regarding the power calculation, a better reference for fall incidence in PwMS is Nilsagård Y, Gunn H, Freeman J, Hoang P, Lord S, Mazumder R, Cameron M. Falls in people with MS--an individual data meta-analysis from studies from Australia, Sweden, United Kingdom and the United States. Mult Scler. 2015 Jan;21(1):92-100. doi: 10.1177/1352458514538884. Epub 2014 Jun 16. PMID: 24948687; PMCID: PMC4361466. This reference includes a much larger number of people, is more recent, and is a meta-analysis, and concludes that ~56% of PwMS fall each 3 months.

I am then a little confused by your use of the term "reduce the fall incidence to 50% in the intervention group" but presume this to mean you will reduce the number of people who fall at least once in 6 months to 50%. However, this does not appear consistent with your proposed statistical analysis plan, although it is hard to tell given the very limited description of your analysis plan for RQ1.

"The International MS Fall Prevention Network recommends that a reduction in falls should be the primary outcome of falls-prevention programs. ... Falls data should be presented as the rate of falls per person per unit time, in addition to the total number of falls and the number of fallers. Information on the consequences of each fall—that is, self-reported injury and health-care use—should also be recorded and reported." Coote S, Sosnoff JJ, Gunn H. Fall Incidence as the Primary Outcome in Multiple Sclerosis Falls-Prevention Trials: Recommendation from the International MS Falls Prevention Research Network. Int J MS Care. 2014 Winter;16(4):178-84. doi: 10.7224/1537-2073.2014-059. PMID: 25694776; PMCID: PMC4321456.

Without any baseline monitoring of falls, how will you detect a reduction of the fall incidence as you seem to define this (i.e. proportion of people in the group who fall over a 6-month period)? I presume in fact what you will do is compare the fall frequency between the active and control group. Please make this clearer in the statistical methods, including whether you will compare changes in fall rates, and if so between which periods. With falls being a continuously collected measurement there are many ways to compare falls incidence between groups. Then decide what is an acceptable difference. Although there is no published MCID for change in fall frequency in PwMS, 25% relative reduction in fall rate has been identified as the MCID in Parkinson's (Emily J. Henderson, Gemma S. Morgan, Jigisha Amin, Daisy M. Gaunt, Yoav Ben-Shlomo, The minimum clinically important difference (MCID) for a falls intervention in Parkinson's: A delphi study, Parkinsonism & Related Disorders,

Volume 61,2019,Pages 106-110,)

I suggest that the discussion be made more robust. If you are tight for word count, the information on the theoretical basis, self-management approach, and pedagogical model could be shortened. Also, this information does not really belong in the methods, it should be either in the introduction as a rationale for your approach or in the discussion.

Consider including "Kannan M, Hildebrand A, Hugos C, Chahine R, Cutter G, Cameron MH. Evaluation of a web-based fall prevention program among people with multiple sclerosis. Multiple Sclerosis and Related Disorders. 2019, 31:151-156." in your introduction and/or discussion as another virtual fall prevention program in PwMS. This was an asynchronous program but with very similar content to your own. Furthermore, the Free From Falls program (your reference 16) is also very similar to your own but unfortunately showed no greater impact on fall frequency than a control intervention of a brochure. I would like to see more discussion of why you expect your program to be more effective. I get the impression that you expect greater success because of the greater engagement of self-management techniques but this should be made explicit. I am concerned you could expend a lot of time and effort to have a similarly negative result.

Per the instructions for protocol studies in BMJ Open, the dates of the study should be included in the manuscript. Please do this.

VERSION 1 - AUTHOR RESPONSE

**Reviewer 1**	
1. Clearly distinguish between patient experience and medical objectives, including how effectiveness and cost-effectiveness are determined.	Thank you for your question. Our primary outcome is fall frequency (RQ1) and secondary outcomes (RQ2) include self-reported data. Our process evaluation (RQ3) includes

	qualitative interviews of patient experiences. In addition, a health economic evaluation will be performed according to standard procedures. We hope that this will make it clearer.
2. Add a hypothesis to the manuscript.	We agree on the importance of hypotheses and have updated the hypothesis in the manuscript.
3. Clarify the participant group—those who have experienced one or more falls in the past year. How does this group differ from those who have never fallen?	Thank you for the question. All participants in the Fewer Falls have fallen in the past year, as this is one of the eligibility criteria described under the heading “Participants”. An important feature of the Fewer Falls program is discussions between participants on previous experiences of falls as a method to learn from each other, wherefore this is an important criterion.<br>Falls occur in both PwMS with mild disability (1) and in those who are non-ambulatory (2). Balance and walking difficulties; fatigue, pain and cognitive impairments; previous falls and fear or concerns of falling are known fall risk factors (2-8).<br>1. Abasıyanık Z, Kahraman T, Ertekin Ö, Baba C, Özakbaş S. Prevalence and determinants of falls in persons with multiple sclerosis without a clinical disability. Mult Scler Relat Disord. 2021;49:102771.<br>2. Abou L, Sosnoff JJ, Peterson EW, Backus D, Willingham TB, Rice LA. Prediction of future falls among full-time wheelchair and scooter users with multiple sclerosis: A prospective study. Mult Scler Relat Disord. 2022;64:103962.<br>3. Gunn HJ, Newell P, Haas B, Marsden JF, Freeman JA. Identification of risk factors for falls in multiple sclerosis: a systematic review and meta-analysis. Phys Ther. 2013;93(4):504-13.

	4. Abou L, McCloskey C, Wernimont C, Fritz NE, Kratz AL. Examination of Risk Factors Associated With Falls and Injurious Falls in People With Multiple Sclerosis: An Updated Nationwide Study. Arch Phys Med Rehabil. 2023.<br>5. Quinn G, Comber L, McGuigan C, Hannigan A, Galvin R, Coote S. Risk factors for falling for people with Multiple Sclerosis identified in a prospective cohort study. Clin Rehabil. 2021;35(5):765-74.<br>6. Cameron MH, Thielman E, Mazumder R, Bourdette D. Predicting falls in people with multiple sclerosis: fall history is as accurate as more complex measures. Mult Scler Int. 2013;2013:496325.<br>7. Gunn H, Creanor S, Haas B, Marsden J, Freeman J. Risk factors for falls in multiple sclerosis: an observational study. Mult Scler. 2013;19(14):1913-22.<br>8. Gunn H, Cameron M, Hoang P, Lord S, Shaw S, Freeman J. Relationship Between Physiological and Perceived Fall Risk in People With Multiple Sclerosis: Implications for Assessment and Management. Arch Phys Med Rehabil. 2018;99(10):2022-9.
4. Clarify whether age is treated as an independent variable.	Thank you. In the analysis, we will adjust for potential confounding factors including e.g., age, health literacy, fatigue, cognition, anxiety and/or depression.
5. Include more literature about online programs, not just regarding falls and the illness condition. For example, refer to studies like https://pubmed.ncbi.nlm.nih.gov/34713024/.	Thank you for the suggestion. We have added the information in the background.
6. The role of the leader who facilitates and motivates participants should be evaluated in real-time. Is this feasible? Additionally, evaluate the impact of a leader on the	We agree on the importance of evaluating the role of the group leader. In our feasibility study (not yet published) we observed all sessions in real-time, interviewed the three

Field Code Changed

effectiveness of online or self-management programs.	group leaders and the participants, and group leaders answered a short questionnaire on the experiences of the session. This evaluation was feasible in our pilot study. However, in the current study we will not observe all sessions due to the risk of affecting the group process. In addition, we decided it to be too time-consuming to observe all sessions. We will, however, as part of the process evaluation (RQ3) evaluate the impact of the facilitators from the perspectives of the group participants. In addition, after each session, the facilitators will answer a short questionnaire on the experiences of the session.
7. Provide more details on the socio-demographic characteristics of the participants.	As this is a study protocol, we do not yet have data on the socio-demographic characteristics. We are however collecting data on the variables age, sex, civil status, work status, and educational level.
8. Update Table 1 to include information on synchronous versus asynchronous meetings, group versus individual settings, and whether a leader is involved.	Thank you for noticing this. We have updated Table 1 with information that the “Sessions” are synchronous, group-based, online and facilitator-led and the “Home assignments” are asynchronous and individual.
9. Clarify the use of models for program evaluation or for analyzing data and outcomes.	Thank you for this question. We have added more information on our statistical analyses plan. As this is a study protocol, we are not yet determined whether to use e.g. SAS or SPSS.
10. How often are participants interviewed during the 8-week period?	Thank you for your question. Participants will not be interviewed during the 8-week period. However, as part of the process evaluation (RQ3) interviews will be conducted at 3, 6 and 12 months after start of the intervention. A purposive selected sample of participants will be interviewed. We have updated the information on participant interviews in RQ3.

11. Discuss whether the cost of the program is covered by health insurance.	The participants have no costs in relation to the program. All costs within the program are covered by the research fundings (see "Funding").
12. Expand the discussion section—it currently reads like a summary and is only a page and a half long.	We understand that it may be confusing with such a short discussion. However, as this is a study protocol we have yet little to discuss. Nonetheless, we have expanded the discussion section to highlight the differences of the Fewer Falls intervention compared to previous RCTs on self-management fall prevention interventions as well as a paragraph with potential limitations.
13. The Results section is missing; what were the key findings?	This is a study protocol wherefore we have no results yet.
14. Clarify the meaning of "ambulatory" and "non-ambulatory" in the context of the ability to walk.	Thank you for highlighting this. We have added information in the "Recruitment and procedures" to clarify the meaning of ambulatory.
15. There is no feasibility survey of online evaluations. Consider including one.	Thank you for the suggestion. In our feasibility study (in manuscript), we identified that 86% of the participants preferred digital questionnaires compared to paper-based questionnaires. The response rate was high, and the participants overall found digital questionnaires easy to use. Our online data collection tool (BASS) is similar to REDCAP, used by for example Kannan 2019. BASS has been used in 50+ RCTs at the Karolinska Institutet, more information can be found at https://ki.se/en/research/research-infrastructure-and-environments/core-facilities-for-research/ehealth-core-facility/online-data-collection
**Reviewer 2**	
1.Please clarify the aims of this study. Are you studying the effectiveness of the intervention and/or its cost-effectiveness? In some places the cost-effectiveness is emphasized (e.g. cost is mentioned in the	Thank you for highlighting this. We have clarified the aims of the study, including a revision of the research questions. The primary aim is to evaluate the effectiveness of the

Field Code Changed

very first paragraph of the introduction and in the first sentence of the aim and research questions) but in other places cost is not mentioned (e.g. the abstract, the overall hypothesis).	*Fewer Falls* self-management fall prevention program on falls in PwMS. The hypothesis is that the fall frequency in the *Fewer Falls* intervention group will be lower compared to the falls frequency in the control group.<br>In addition to evaluation of effectiveness (RQ 1 and 2) we perform a process evaluation (RQ 3) and health economic evaluation (RQ 4).
2. I find the hypothesis as stated, "that after participating in Fewer Falls, PwMS will identify and self-manage risk factors for falls in their everyday life, and thereby reduce the number of falls." somewhat unclear and difficult to test. This hypothesis would require four things to be true: 1) PwMS identify their fall risk factors in everyday life, 2) self-manage fall risk factors in everyday life, 3) reduce their number of falls, 4) that 1 and 2 are causative of 3. Furthermore, the research questions (RQ) do not directly address these issues. RQ1 addresses the number of falls, i.e. #3. 1, 2 and 4 are not addressed by the RQs, and then the RQs address completely different things i.e. secondary outcomes (RQ2), process evaluation components (RQ3), and cost effectiveness (RQ4).	Thank you for these valuable comments. We have thoroughly revised and clarified our hypothesis, aims, and research questions, as presented under the heading “Aim and research questions”. We hope this will make it clear.
3. It is not clear to me if this study is ongoing or planned at this time.<br><br>Either way, I think one could align the aims, hypothesis(es), and research questions with each other in this manuscript. If the study is planned it could probably be improved. If it is ongoing one can still improve this description.	The study is ongoing. The recruitment began in December 2023, and expected completion of the study is January 2027. We have added information in “Recruitment and Procedures”.<br><br>We hope you find that the description of the study has improved in the revised manuscript where we have aligned the hypothesis, aims, and research questions with each other.
4. https://ki-se.zoom.us/j/63115732144https://ki-se.zoom.us/j/63115732144https://ki-se.zoom.us/j/63115732144https://ki-	Thank you for this comment. We agree on the importance of a detailed description. As a very detailed description of the program

se.zoom.us/j/63115732144In the methods please add some specific details regarding patient and public involvement. You state that "the intervention has been developed in close collaboration with PwMS and healthcare professionals." Please be specific e.g. the intervention was developed initially by the investigators based on their background knowledge and current literature. An initial version of the intervention was presented to a focus group of 5 PwMS (some demographics about them) and 3 healthcare professionals (a nurse, a PT and an OT). Based on this input the intervention was modified ... The readership needs this level of detail to understand the degree of influence of various parties.	development is already published, we prefer referring to this publication in the manuscript under the heading "Patient and Public Involvement". Link to the publication: Development of Fewer Falls in MS-An Online, Theory-Based, Fall Prevention Self-Management Programme for People With Multiple Sclerosis - PubMed (nih.gov)
5. Regarding the participants, how did you/will you verify they have MS? or that they have had one or more falls in the past year?	Thank you for this question. Unfortunately, it is not possible for us to have a physician to physically examine and verify the diagnosis as we recruit from whole Sweden. However, our research assistant will screen all participants for eligibility, including that participants report having MS diagnosis and having experienced a previous fall during the last year. It is in Sweden very unlikely that a person would claim that they have MS without having been diagnosed. As for previous falls, we rely on what interested participants report which is a common approach in fall prevention research.
Regarding the power calculation, a better reference for fall incidence in PwMS is Nilsagård Y, Gunn H, Freeman J, Hoang P, Lord S, Mazumder R, Cameron M. Falls in people with MS--an individual data meta-analysis from studies from Australia, Sweden, United Kingdom and the United States. Mult Scler. 2015 Jan;21(1):92-100. doi: 10.1177/1352458514538884. Epub 2014 Jun 16. PMID: 24948687; PMCID: PMC4361466. This reference includes a much larger number of people, is more	Thank you very much for this comment. We agree that the proposed reference is much better than the one we used and have added this reference to the Introduction. The power calculation is revised and is now based on data from our pilot RCT on fall frequency (primary outcome in the full-scale RCT) as described under the heading "Participants".

Field Code Changed

recent, and is a meta-analysis, and concludes that ~56% of PwMS fall each 3 months.	
6. I am then a little confused by your use of the term "reduce the fall incidence to 50% in the intervention group" but presume this to mean you will reduce the number of people who fall at least once in 6 months to 50%. However, this does not appear consistent with your proposed statistical analysis plan, although it is hard to tell given the very limited description of your analysis plan for RQ1.	Thank you for this valuable comment and we do agree. We acknowledge that it has been unclear and confusing but hope that the revised manuscript will make it clearer. We are going to evaluate if there is a difference in fall frequency between groups. Our hypothesis is that fall frequency, i.e., falls/person/year, will be lower in the intervention group compared to the one in the control group. Our statistical plan has also been up-dated and we hope that you find it satisfactory.
7. "The International MS Fall Prevention Network recommends that a reduction in falls should be the primary outcome of falls-prevention programs. ... Falls data should be presented as the rate of falls per person per unit time, in addition to the total number of falls and the number of fallers. Information on the consequences of each fall—that is, self-reported injury and health-care use—should also be recorded and reported." Coote S, Sosnoff JJ, Gunn H. Fall Incidence as the Primary Outcome in Multiple Sclerosis Falls-Prevention Trials: Recommendation from the International MS Falls Prevention Research Network. Int J MS Care. 2014 Winter;16(4):178-84. doi: 10.7224/1537-2073.2014-059. PMID: 25694776; PMCID: PMC4321456.	Thank you. We do agree and we have in the revised version of the manuscript clarified that we have falls as primary outcome, presented as falls/person/year. We will of course also document and report the total number of falls, number of fallers, and the consequences of each fall.
8. Without any baseline monitoring of falls, how will you detect a reduction of the fall incidence as you seem to define this (i.e. proportion of people in the group who fall over a 6-month period)? I presume in fact what you will do is compare the fall frequency between the active and control group. Please make this clearer in the statistical methods, including whether you will compare changes in fall rates, and if so between which periods. With falls being a continuously collected measurement there	Again, thank you very much for your valuable comments. You are perfectly right that we will compare fall frequency between groups. We have revised the manuscript accordingly and hope you will find it clear how we plan to analyze primary and secondary outcomes.<br><br>Thank you for making us aware of the interesting Delphi study.

are many ways to compare falls incidence between groups.<br><br>9. Then decide what is an acceptable difference. Although there is no published MCID for change in fall frequency in PwMS, 25% relative reduction in fall rate has been identified as the MCID in Parkinson's (Emily J. Henderson, Gemma S. Morgan, Jigisha Amin, Daisy M. Gaunt, Yoav Ben-Shlomo, The minimum clinically important difference (MCID) for a falls intervention in Parkinson's: A delphi study, Parkinsonism & Related Disorders,<br>Volume 61,2019,Pages 106-110,)	We agree that it is difficult to decide what constitutes a clinically relevant change. We have no baseline data and will therefore analyze differences between groups. In our pilot feasibility study the calculated fall/person/year was 11 and 7 for the control and intervention group, respectively. We have decided that the difference of 4 fall/person/year between groups could be considered as a clinically important difference. This difference exceeds the 25% relative reduction reported in the delphi study.
10. I suggest that the discussion be made more robust. If you are tight for word count, the information on the theoretical basis, self-management approach, and pedagogical model could be shortened. Also, this information does not really belong in the methods, it should be either in the introduction as a rationale for your approach or in the discussion.<br>Consider including "Kannan M, Hildebrand A, Hugos C, Chahine R, Cutter G, Cameron MH. Evaluation of a web-based fall prevention program among people with multiple sclerosis. Multiple Sclerosis and Related Disorders. 2019, 31:151-156." in your introduction and/or discussion as another virtual fall prevention program in PwMS. This was an asynchronous program but with very similar content to your own. Furthermore, the Free From Falls program (your reference 16) is also very similar to your own but unfortunately showed no greater impact on fall frequency than a control intervention of a brochure. I would like to see more discussion of why you expect your program to be more effective. I get the impression that you expect greater success because of the greater engagement of self-management techniques but this should be made explicit. I am concerned you could expend a lot of time and effort to have a similarly negative result.	Thank you for this comment.<br>We have chosen to include the information on the theoretical basis, self-management approach, and pedagogical model in the method section as we believe this information is important for the understanding of the intervention.<br><br>Thank you for this comment. We have included the Kannan reference in the introduction as an example of an online program. Although there are similarities there are also important differences since the Fewer Falls program has a clear focus on self-management and behavioral change to address the participants individual fall risks. To clarify we have added information in the intervention description and a paragraph in the discussion section.

Formatted: Font: (Default) Aptos, English (United Kingdom)

11. Per the instructions for protocol studies in BMJ Open, the dates of the study should be included in the manuscript. Please do this.	We have added information regarding recruitment start and expected completion of the study in "Recruitment and Procedures".

Formatted: Font: (Default) Aptos, English (United Kingdom)

VERSION 2 - REVIEW

**Reviewer**	**2**
**Name**	**Cameron, Michelle**
**Affiliation**	**Oregon Health & Science University**
**Date**	**18-Nov-2024**
**COI**	

Thank you for addressing my feedback so thoroughly. This is now a much improved manuscript. I have a few minor requests or suggestions.

1. In the section on participants, this would read more clearly if the participant description was written in terms of inclusion and exclusion criteria. As written, it is unclear if you have any exclusion criteria. You could then define PwMS in inclusion criteria as "self-reported diagnosis of MS". This is fine. You don't need physician confirmation, you just need to be clear about how these people were selected. Writing this way would allow for study replication if desired. Additionally, are you able to convert your disability related inclusion criterion to an EDSS or PDDS level, again for more ready comparison to other trials. This may or may not be possible.

2. Allowing for a 35% dropout is unusually high. Please address this in the discussion. Why do you expect such high dropout? What does this say about the acceptability and feasibility of the program, and of the study?

3. Please make the discussion more robust. I realize you cannot discuss any results but the approach has strengths and limitations. You have focused on the strengths. Limitations include, but are not limited to, the following: this study is only in Sweden, it is limited to those with the technical and cognitive ability to participate in a fairly complex program, my guess is that sociodemographics will be limited (e.g. race, level of education, income level, maybe also rurality), the high expected drop out rate. What other limitations do you know up front? all studies have them, that's why we do so many studies of the same thing.

4. One more edit of the text to improve the English would be helpful. Although everything can be understood, the paper would be read more easily if the English language were a little cleaner.

VERSION 2 - AUTHOR RESPONSE

**Reviewer: 2**	
1. In the section on participants, this would read more clearly if the participant description was written in terms of inclusion and exclusion criteria. As written, it is unclear if you have any exclusion criteria. You could then define PwMS in inclusion criteria as "self-reported diagnosis of MS". This is fine. You don't need physician confirmation, you just need to be clear about how these people were selected. Writing this way would allow for study replication if desired. Additionally, are you able to convert your disability related inclusion criterion to an EDSS or PDDS level, again for more ready comparison to other trials. This may or may not be possible.	Thank you for these valuable suggestions. The participant description is now written in terms of inclusion criteria, and 'self-reported diagnosis of MS' is added. The disability related inclusion criterion corresponds to, up to 7.5 on the EDSS, which is added in the text.
2. Allowing for a 35% dropout is unusually high. Please address this in the discussion. Why do you expect such high dropout? What does this say about the acceptability and feasibility of the program, and of the study?	Thank you for this suggested clarification. We have addressed this in the methods. Digital self-management programs in chronic diseases display high attrition and drop-out rates. Thus, to be confident that we during our 6- and 12-months follow-ups have enough power, we will have the possibility to include up to 240 PwMS. We will explore the acceptability of the program in the process evaluation.
3. Please make the discussion more robust. I realize you cannot discuss any results but the approach has strengths and limitations. You have focused on the strengths. Limitations include, but are not limited to, the following: this study is only in Sweden, it is limited to those with the technical and cognitive ability to participate in a fairly complex program, my guess is that sociodemographics will be limited (e.g. race, level of education, income level, maybe also rurality), the high expected drop-out rate. What other	Thank you so much for this comment. We have added text to the discussion on limitations based on your suggestions.

limitations do you know up front? all studies have them, that's why we do so many studies of the same thing.	
4. One more edit of the text to improve the English would be helpful. Although everything can be understood, the paper would be read more easily if the English language were a little cleaner.	Thank you for this comment. Our native speaking co-author has edited the manuscript.
